# Trends in diversity of scientific authorship by ethnicity, sex and age of authors in a National Institute for Health and Care Research Biomedical Research Centre in England: a retrospective longitudinal bibliometric study

**DOI:** 10.1136/bmjopen-2025-115677

**Published:** 2026-10-15

**Authors:** Lorna R Henderson, F Eugenio Barrio, David Saunders, Owen Coxall, Vasiliki Kiparoglou, Evanthia Kalpazidou Schmidt, Syed Ghulam Sarwar Shah

**Affiliations:** 1 NIHR Oxford Biomedical Research Centre, Oxford University Hospitals NHS Foundation Trust, Oxford, England, UK; 2 Radcliffe Department of Medicine, University of Oxford, Oxford, England, UK; 3 Research Services, University of Oxford, Oxford, England, UK; 4 Bodleian Health Care Libraries, University of Oxford, Oxford, England, UK; 5 Northwick Park & St Mark’s Hospital, The Griffin Institute, London, UK; 6 Department of Political Science, Danish Centre for Studies in Research and Research Policy Aarhus University, Aarhus, Denmark; 7 Department of Natural Sciences, Middlesex University, London, UK

**Keywords:** Health policy, Health Workforce, Observational Study, Workplace, Cross-Sectional Studies

## Abstract

**Abstract:**

**Objective:**

Scientific publications are important markers of achievement in academic medicine. While gender disparities in scientific authorship are well documented, there is little understanding of the influence of authors’ protected characteristics on authorship positions, publication outputs and citation counts in biomedical and translational health research. This study examined the diversity of scientific authorship by three protected characteristics, that is, ethnicity, sex and age, of authors of peer-reviewed biomedical and translational health research publications.

**Design:**

A baseline feasibility study that involved retrospective longitudinal bibliometric analysis of 476 unique authors’ protected characteristics, peer-reviewed publication outputs and citation counts from 2012 to 2022.

**Setting:**

National Institute for Health and Care Research (NIHR) Oxford Biomedical Research Centre (BRC) in England.

**Data:**

Peer-reviewed publications funded by the NIHR Oxford BRC during its funding round 2 (2012–2017) (henceforth BRC2) and round 3 (2017–2022) (henceforth BRC3). BRC author-level data were extracted on three self-reported protected characteristics, that is, ethnicity, sex (male and female) and age of authors. Data were analysed by descriptive statistics, Fisher’s exact test and Pearson’s χ^2^ test of independence.

**Primary outcome measures:**

Authorship positions (first and last authors), publication outputs (number of publications) and citation counts (number of citations) of peer-reviewed publications by the author’s ethnicity, sex and age.

**Results:**

White researchers were 79% and 85% of first authors and 85% and 86% of last authors in the BRC2 and BRC3 periods, respectively. In the first author category, females were the majority (53%) in the BRC2 period, while males were in the majority (59%) in the BRC3 period (p=0.014). In the last author category, males were more (70% and 78%) than females (30% and 22%) in the BRC2 and BRC3 periods, respectively (p<0.001). In the total study period (2012–2022), female first (44%) and last authors (24%) were statistically significantly less than male first (56%) and last (76%) authors (p<0.001). Authors aged 40–59 years were statistically significantly more (68% and 61% of first authors and 84% and 75% of last authors in BRC2 and BRC3, respectively) than authors of other age groups (p<0.001).

White authors published more publications (87%) than Black, Asian and Minority Ethnic (BAME) authors (13%) in the BRC2, BRC3 and total study period. There were no differences in the overall proportions of publications by authors of either ethnicity in the BRC2 and BRC3 periods (p=0.91). Male authors produced statistically significantly more publications (59% and 73%) than female authors (41% and 27%) in the BRC2 and BRC3 periods, respectively (p<0.001). Authors aged 40–59 years published more papers (64.5% and 69% of total papers in the BRC2 and BRC3 periods, respectively) than authors of other age groups (p<0.001).

Citation counts of publications by white authors were statistically significantly higher (89% and 87% of total citation counts in the BRC2 and BRC3 periods, respectively) than citation counts of publications by BAME authors (p<0.001). Publications by male authors had higher citation counts (65% and 68% of total citation counts in the BRC2 and BRC3 periods, respectively) than citation counts of female authors’ publications (p<0.001). Citation counts of publications by authors aged 40–59 years were higher (56% and 71% of total citation counts in the BRC2 and BRC3 periods, respectively) than citation counts of publications by authors aged less than 40 years and those aged 60 and above (p<0.001). Authors aged 60–64 years had the highest average citations per publication in both BRC periods and the total study period. Some positive trends were observed in the BRC3 period compared with the BRC2 period, such as a substantial increase in the absolute number of BAME authors, female (first and last) authors, and young first authors (aged up to 39 years), and an increase in the number of publications by BAME, female and young authors. In addition, female authors’ publications had higher average citations (42 citations per publication) compared with male authors’ publications (33 citations per publication) in the BRC3 period.

**Conclusion:**

There are statistically significant differences in authorship positions, publication outputs and citation counts by ethnicity, sex and age of authors affiliated with the NIHR Oxford BRC. Although some encouraging trends are noted, there is under-representation of authors from BAME backgrounds, women and those under 40 years of age. Understanding the determinants of these gaps requires systematic collection of data on protected characteristics of authors and researchers in BRC settings.

**STRENGTHS AND LIMITATIONS OF THIS STUDY**

- In this baseline feasibility study, we examined authorship diversity by exploring the influence of the author’s three protected characteristics, that is, ethnicity, sex and age, on three important publication metrics, that is, authorship positions, publication outputs and citation counts within the context of a National Institute for Health and Care Research Biomedical Research Centre (NIHR BRC) in England.
- Benchmarking of protected characteristics data at the individual author level is not routinely available or collected, and completion of this data is self-reported and not mandatory; and hence it is incomplete.
- We used novel data capture techniques, which included research publication data captured via the Symplectic Elements research database and linked it with the University of Oxford’s core human resources data on self-reported protected characteristics data of researchers affiliated with the NIHR Oxford BRC.
- Although our innovative approach provides benchmarking data on scientific authorship diversity of relevance to NIHR funded BRCs, further work is needed to improve the reliability and comprehensiveness of these data.

## Introduction

 Biomedical Research Centres (BRCs) are translational research organisations established in England in 2007 by the National Institute for Health and Care Research (NIHR).^1^ BRCs are partnerships between leading National Health Service (NHS) hospital trusts and universities, and they are required to track equality, diversity and inclusion (EDI) improvements as a prerequisite for translational research funding.^2 3^ Peer-reviewed scientific publications are markers of achievement and determinants of career progression.^4 5^ Citations of publications are also important markers of success, impact and quality.^6–8^ Contractually, the number of peer-reviewed publications by BRC-supported researchers (authors) is reported to the funder, that is, the NIHR.^9^ However, little is understood about the diversity of the scientific authorship of research funded by the NIHR BRCs.

Authorship position is an important marker of achievement, with ‘first author’ and ‘last author’ positions being the most highly regarded. The first author position denotes the author has completed the majority of the work, and it is an important marker to enhance career development.^10^ The last (senior) author position is typically given to senior academics, leads and group leads, and it is an important marker of achievement and demonstrates group leadership and funding oversight.^11^

### Study aim and objectives

The aim was to conduct a baseline feasibility study on the diversity in scientific authorship by the author's three protected characteristics, that is, ethnicity, sex and age. Previous research has examined these factors individually, such as equity in authorship by the author’s sex/gender^1 5 9 12^ or ethnicity.^13 14^

The objective was to explore the influence of the author’s ethnicity, sex and age on authorship positions (first and last authors), publication outputs (number of publications) and citation counts (number of citations) of peer-reviewed biomedical and translational health research publications.

## Methods

### Study design

This baseline feasibility study involved a retrospective longitudinal bibliometric analysis of peer-reviewed biomedical and translational health research publications published between 2012 and 2022.

### Setting

This study was conducted at the NIHR Oxford BRC, which is a research collaboration between the Oxford University Hospitals NHS Foundation Trust and the University of Oxford.^15^ The NIHR BRCs support translational research and innovation to improve healthcare for patients.^16^ Our BRC was one of the first BRCs funded by the NIHR in 2007 when it received its first competitive research grant award of £57 million to support translational health research for 5 years (2007–2012). In 2012, the BRC received a second funding award of £95.5 million for another 5 years (2012–2017) for research across nine research themes and five cross-cutting themes. Thereafter, in 2016, our BRC was awarded £113.7 million funding for the third 5-year period (2017–2022) to support research in 20 research themes.^15^

### Data

Data comprised (a) peer-reviewed publications published by 476 unique researchers affiliated with the NIHR Oxford BRC and (b) the researchers’ (authors’) self-reported protected characteristics data on ethnicity (white and BAME (Black, Asian and Minority Ethnic)), legal sex (male/female) and age (in 5-year groups).

Our BRC funded research authors (researchers) are recorded on Symplectic Elements (‘Elements’), which is the current research information system (CRIS) of the University of Oxford. The Elements database holds records of researchers’ publications and other research activity and can report by researchers’ BRC research theme for the purposes of annual reporting to the funder NIHR, which is a contractual requirement. The eligibility criteria included the researcher recorded in Elements as a member of BRC theme(s) and at least one peer-reviewed journal article linked to their account in Elements.

Data extracts of 476 distinct BRC researchers and their affiliation to themes, publication lists (including full bibliographic and bibliometric metadata) and authorship positions (first and last authors) were downloaded from the Elements database for two BRC research funding periods, that is, 2012–2017 (BRC2) and 2017–2022 (BRC3). The team at Research Services, responsible for the Elements database, liaised with the University of Oxford Human Resources (HR) Department, who independently extracted data on researchers’ three self-reported protected characteristics: that is, legal sex (male/female), ethnicity (white and BAME) and age from the HR database (CoreHR) of the University of Oxford. Data were provided in aggregate form and anonymously to ensure individual researchers were not identifiable. The process of data capture, linking and analysis is illustrated in figure 1.

**Figure 1:**
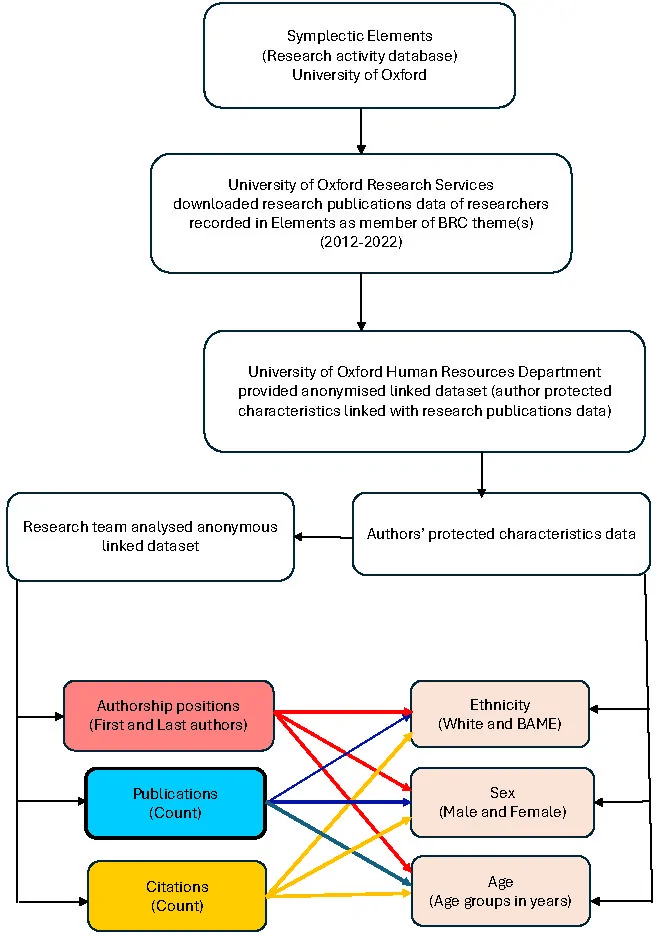
Data capture, linking and analysis process. BAME, Black and Asian Minority Ethnic; BRC, Biomedical Research Centre.

### Outcome measures

The key outcome measures were three publication metrics: authorship positions (first and last authors), publication outputs (number of publications) and citation counts (number of citations) of publications by three protected characteristics of authors: ethnicity (white and BAME), sex (male and female) and age (18 years and above in 5-year age groups).

The outcome measures were compared for two BRC research funding periods, that is, 2012–2017 (BRC2) and 2017–2022 (BRC3). The first funding period (2007–2012, BRC1) was not analysed because the relevant data were not collected for that period.

### Statistical analysis

Data were analysed by descriptive statistics, Fisher’s exact test and Pearson’s χ^2^ test of independence using IBM SPSS Statistics, V.28 for Windows (Armonk, NY: IBM Corp). In addition, percentage changes in publications and citations in the BRC3 period compared with the BRC2 period by author ethnicity, sex and age were calculated using Microsoft Excel, which was also used for creating visualisations.

We calculated percentage changes in publications and citations in the BRC3 period compared with the BRC2 period by using the following formula:

$Change\;in\;publications\;or\;citations\;in\;BRC3=\frac{Total\;publications\;\left(or\;citations\right)\;in\;BRC3-\;Total\;publications\;\left(or\;citations\right)\;in\;BRC2}{Total\;publications\;\left(or\;citations\right)\;in\;BRC2}\;X\;100$

We also calculated average citations per publication by author ethnicity, sex and age by a formula as under:

$Average\;citations\;per\;publication=\frac{Total\;citations\;of\;total\;publications\;in\;BRC2\;(or\;BRC3)}{Total\;publications\;in\;BRC2\;(or\;BRC3)}$

### Patient and public involvement

There was no patient or public involvement in the study design.

## Results

### Authorship positions by ethnicity, sex and age of authors

Proportions of authorship positions by ethnicity, sex and age of authors are presented in table 1. White authors’ proportions were higher in both first and last authorship positions compared with BAME authors in both the BRC2 and BRC3 periods. There were no statistically significant differences in the total proportions of white and BAME first and last authors between the BRC2 and BRC3 periods as well as for the total study period (combined BRC2 and BRC3 periods=2012–2022) (table 1).

**Table 1:** Authorship positions by ethnicity, sex and age of authors

	Authorship positions								
Author characteristics	BRC2 (2012–2017)	BRC3 (2017–2022)		BRC2 (2012–2017)	BRC3 (2017–2022)		Total study period (2012–2022)		
	First authors	First authors	P value	Last authors	Last authors	P value	First authors	Last authors	P value
	Count (%)	Count (%)		Count (%)	Count (%)		Count (%)	Count (%)	
Ethnicity									
BAME	30 (21)	63 (15)	0.111*	69 (15)	182 (14)	0.587*	93 (16)	251 (14)	0.179*
White	115 (79)	357 (85)		393 (85)	1128 (86)		472 (84)	1521 (86)	
Sex									
Female	78 (53)	201 (41)	**0.014** *	147 (30)	350 (22)	**<0.001** *	279 (44)	497 (24)	**<0.001** *
Male	70 (47)	287 (59)		344 (70)	1217 (78)		357 (56)	1561 (76)	
Age group									
Up to 39 years	17 (11.5)	104 (22)	**<0.001** †	41 (8)	40 (3)	**<0.001** †	121 (19.5)	81 (4)	**<0.001** †
40–49 years	47 (32)	156 (33)		270 (56)	418 (27)		203 (32.8)	688 (34)	
50–59 years	53 (36)	130 (28)		139 (28)	741 (48)		183 (29.6)	880 (43)	
60–64 years	17 (11.5)	67 (14)		25 (5)	193 (13)		84 (13.6)	218 (11)	
65 years and above	14 (9)	14 (3)		16 (3)	143 (9)		28 (4.5)	159 (8)	

Statistically significant p-values (p<0.05; <0.01 and <0.001) are shown in bold.

* Fisher’s exact test.

† Pearson’s χ2 test of independence.

BAME, Black, Asian and Minority Ethnic; BRC, Biomedical Research Centre.

Authorship positions by author sex (male and female) showed statistically significant disparities (table 1). Female first authors’ proportion was higher than male first authors’ proportion in the BRC2 period, while female first authors’ proportion was lower than male first authors’ proportion in the BRC3 period (p=0.014). In the last authorship category, male authors’ proportion was statistically significantly greater than female authors’ proportion in both the BRC2 and BRC3 periods (p<0.001) as well as in the total study period (p<0.001).

Authorship positions by author age (table 1) also showed statistically significant disparities in proportions of first and last authors of different age groups (p<0.001). In the BRC2 period, authors aged 50–59 years comprised the highest proportion of first authors, while in the BRC3 period, authors aged 40–49 years made up the highest proportion. In the BRC2 period, the majority of last authors were aged 40–49 years, while in the BRC3 period, the majority of last authors were aged 50–59 years. The proportion of first authors aged 39 and younger increased in the BRC3 period compared with the BRC2 period, while their proportion as last authors decreased in the BRC3 period compared with the BRC2 period. For the total study period (2012–2022), there were statistically significant differences in the proportions of first and last authors by age of authors (p<0.001). The highest proportions of first and last authors were of authors in the age groups 40–49 and 50–59 years, respectively, in the total study period (table 1).

Authorship positions by author age (figure 2—panel A) showed that the proportion of first authors was substantial at the younger age (less than 40 years), peaked in the 40s, and declined during the 50s and onwards. Conversely, the proportion of last authors was very small at the younger age (less than 40 years), increased during the 40s, peaked in the 50s, and then declined in the 60s and onwards (figure 2—panel A). Interestingly, there was a clear divide in first and last authorship positions by age when the author age was divided into two age groups: (a) 49 years and below, and (b) 50 years and above (figure 2—panel B). Authors aged 49 years and below comprised the highest proportion of first authors and the lowest proportion of last authors, while authors aged 50 years and above comprised the highest proportion of last authors and the lowest proportion of first authors; however, the differences were not statistically significant (p=0.064).

**Figure 2:**
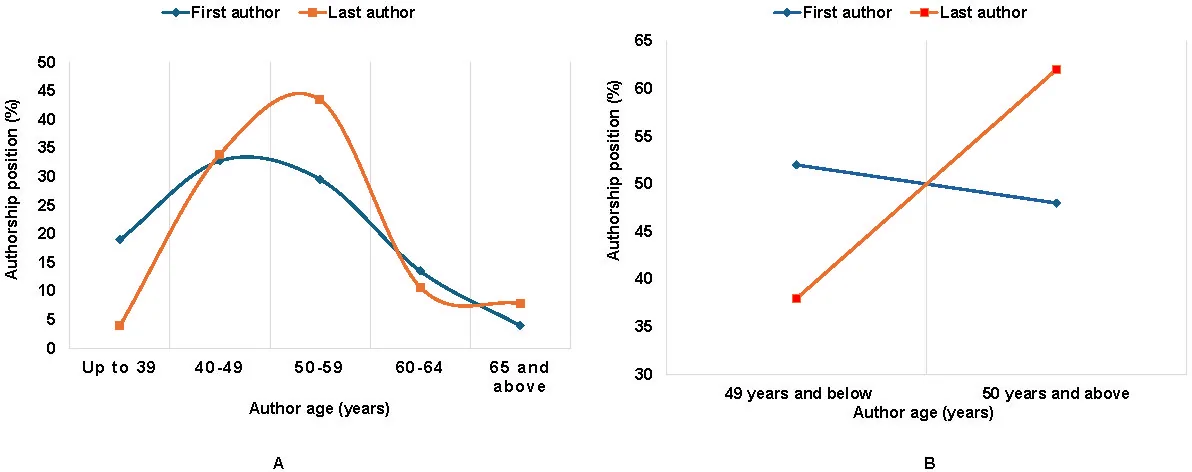
First and last authorship positions by author age (total study period 2012–2022).

The percentage change in authorship positions in the BRC3 period compared with the BRC2 period (figure 3) showed a substantial increase in the first author position by ethnicity, sex and age of authors except for authors aged 65 years and above. In the BRC3 period, there was also a notable increase in the last author position by ethnicity, sex and age for all authors except for authors under 39 years of age, who decreased by 2.4% compared with the BRC2 period (figure 3).

**Figure 3:**
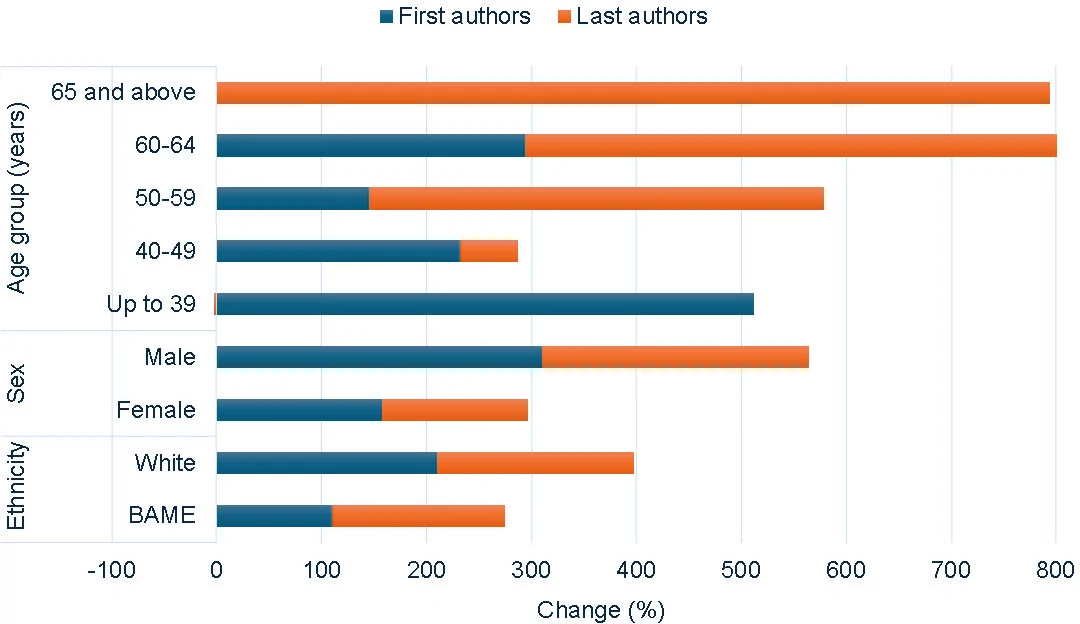
Percentage change in authorship positions by ethnicity, sex and age of authors in BRC3 compared with BRC2. BAME, Black, Asian and Minority Ethnic.

### Publication outputs by ethnicity, sex and age of authors

Publication outputs by author ethnicity, sex and age are presented in table 2, which shows disparities between authors of different ethnic backgrounds, sexes and age groups. Publication outputs by white authors were about nine times higher than publication outputs by BAME authors in both BRC periods, and the overall proportions of publication outputs by author ethnicity were the same (white authors 87% and BAME authors 13%) in both the BRC2 and BRC3 periods (p=0.91). Male authors’ publication outputs were statistically significantly more than female authors’ publication outputs in both BRC periods (p<0.001). Proportions of publication outputs by authors of different age groups were statistically significantly different in both BRC periods (p**<**0.001). Publication outputs by authors aged 50–59 years were more than publication outputs by authors of any other age group in both BRC periods. Irrespective of the ethnicity, sex and age of authors, the total publication output substantially increased in the BRC3 period compared with the BRC2 period (table 2). In the total study period (2012–2022), authors who were white, male and 50–59 years of age produced the highest proportions of publications compared to authors of any other ethnicity, sex or age group (table 2).

**Table 2:** Publications and citations by ethnicity, sex and age of authors

Author characteristics	Publications				Citations			
	BRC2 (2012–2017)	BRC3 (2017–2022)	P value	Total study period (2012–2022)	BRC2 (2012–2017)	BRC3 (2017–2022)	P value	Total study period (2012–2022)
	Count (%)	Count (%)		Count (%)	Count (%)	Count (%)		Count (%)
Ethnicity								
BAME	263 (13)	723 (13)	0.91*	986 (13)	18 439 (11)	26 761 (13)	**<0.001** *	45 200 (12)
White	1777 (87)	4835 (87)		6612 (87)	149 341 (89)	172 951 (87)		322 292 (88)
Sex								
Female	891 (41)	1792 (27)	**<0.001** *	2683 (31)	62 217 (35)	75 516 (32)	**<0.001** *	137 733 (34)
Male	1285 (59)	4816 (73)		6101 (69)	113 646 (65)	158 948 (68)		272 594 (66)
Age group								
Up to 39 years	72 (3.3)	461 (7)	**<0.001** *	533 (6)	5541 (2)	13 595 (6)	**<0.001** *	19 136 (3)
40–49 years	399 (18.3)	1736 (27)		2135 (25)	55 122 (17)	73 590 (33)		128 712 (23)
50–59 years	1005 (46.2)	2731 (42)		3736 (43)	129 299 (39)	87 097 (38)		216 396 (39)
60–64 years	436 (20.1)	938 (15)		1374 (16)	97 050 (29)	40 568 (18)		137 618 (25)
65 years and above	263 (12.1)	564 (9)		827 (10)	43 578 (13)	11 112 (5)		54 690 (10)

Statistically significant p-values (p<0.05; <0.01 and <0.001) are shown in bold.

* Pearson’s χ2 test of independence.

BAME, Black, Asian and Minority Ethnic; BRC, Biomedical Research Centre.

The percentage change in the number of publications by ethnicity, sex and age of authors in the BRC3 period compared with the BRC2 period is shown in figure 4. Publications by BAME authors increased by 175% compared with the 172% increase in white authors’ publications. Publications by male authors increased by 275%, while female authors’ publications increased by 101%. Very interestingly, the percentage change in publications by young authors aged up to 39 years was the highest (540%) among authors of all age groups (figure 4).

**Figure 4:**
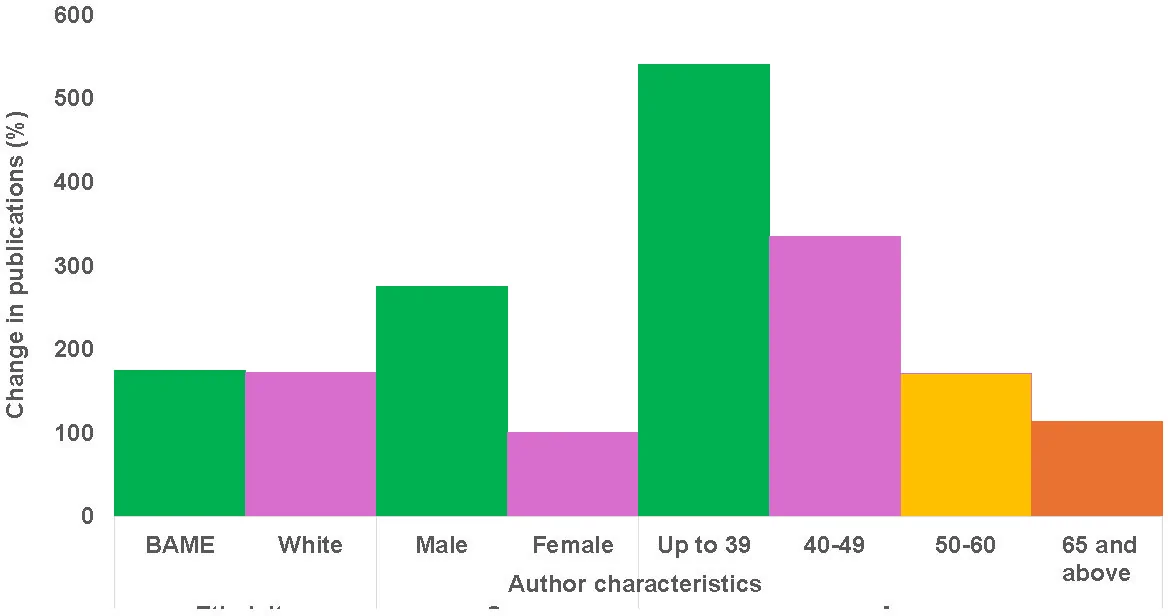
Percentage change in publications by ethnicity, sex and age of authors in BRC3 compared with BRC2. BAME, Black, Asian and Minority Ethnic.

### Citation counts by ethnicity, sex and age of authors

Citation counts by ethnicity, sex and age of authors were statistically significantly different in the BRC2 and BRC3 periods (p<0.001), as shown in table 2. The highest citation counts were for publications by authors who were white, male and 50–59 years of age in BRC2, BRC3 and the total study period (table 2). The absolute number of citations of publications of the BRC3 period was higher than the citations of publications of the BRC2 period by all authors irrespective of their ethnicity, sex and age except for authors who were 50 years and above in age (table 2).

The percentage change in total citation counts in the BRC3 period compared with the BRC2 period (figure 5) showed that citation counts of publications by BAME authors increased more (45%) than citation counts of publications by white authors (16%), and citation counts of publications by male authors increased more (40%) than citation counts of publications by female authors (21%). Total citation counts of publications by authors aged up to 39 years and aged 40–49 years increased by 145% and 33%, respectively; however, citation counts of publications by authors aged 50–59 years, 60–64 years, and 65 years and above decreased by 33%, 58% and 75%, respectively, in the BRC3 period compared with the BRC2 period (figure 5).

**Figure 5:**
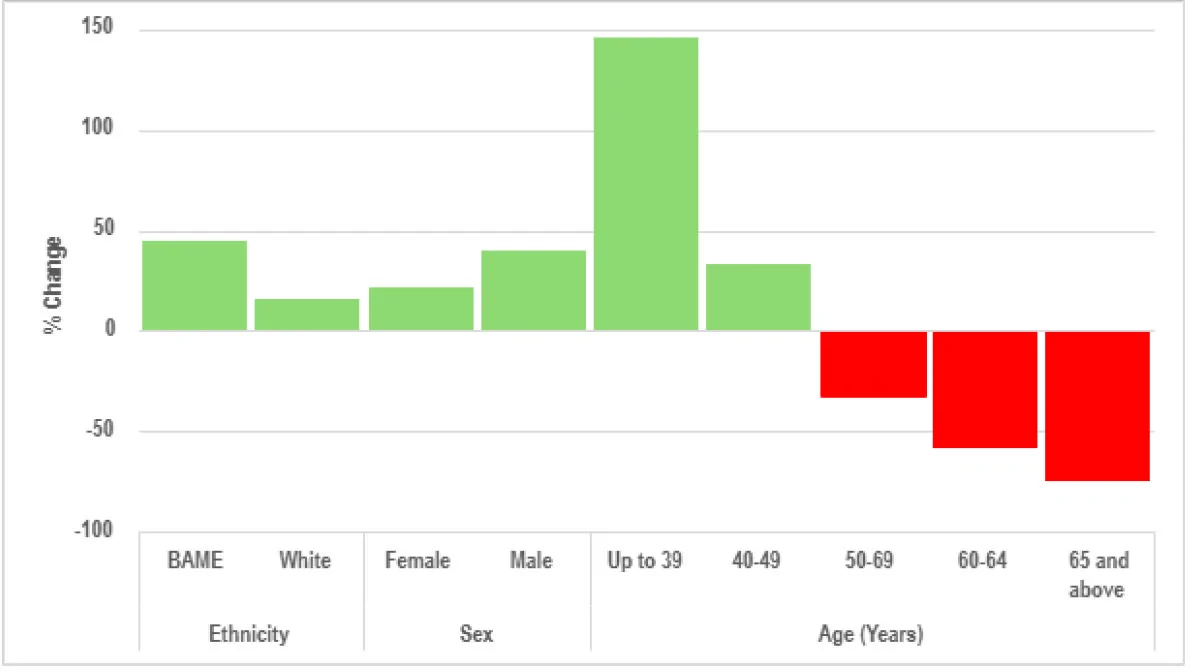
Percentage change in total citations by ethnicity, sex and age of authors in BRC3 compared with BRC2. BAME, Black, Asian and Minority Ethnic.

Analysis of average citations per publication in the BRC2 period showed that authors who were white, male and 60–64 years of age had the highest citations compared with authors of other ethnicities, sexes and age groups (figure 6). BRC2 publications by white authors received higher average citations (84 citations per publication) compared with publications by BAME authors (70 citations per publication). However, BRC3 publications by BAME authors were cited slightly more than publications by white authors (37 vs 36 citations per publication, respectively) (figure 6). In addition, BRC3 publications by female authors had higher average citations per publication than male authors’ publications (42 vs 33 citations per publication, respectively) (figure 6). Average citations per publication over the total study period (2012–2022) revealed that female authors’ publications had higher average citations than male authors’ publications (51 vs 45 citations per publication, respectively) (figure 6). In addition, publications by authors aged 60–64 years had the highest average citations per publication in BRC2, BRC3, and the total study period (figure 6).

**Figure 6:**
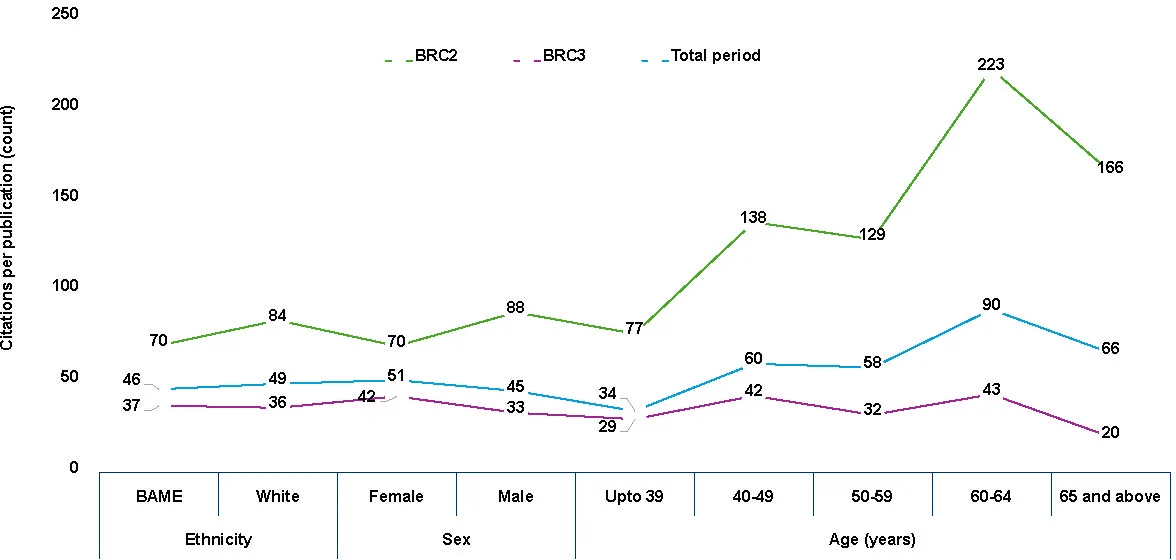
Average citations per publication by ethnicity, sex and age of authors. BAME, Black and Asian Minority Ethnic.

### Discussion

We retrospectively analysed biomedical and translational health research publications funded by the NIHR Oxford BRC between April 2012 and November 2022. We analysed research publications at an individual researcher level, as it is the most common unit of analysis.^17 18^ We did this study at the research environment level, that is, at the level of our BRC providing research facilities and funds,^19^ which are crucial factors in promoting research productivity^20 21^ and fostering research collaborations.^22^ Our BRC is a large research infrastructure that supports innovative and translational health research. Latest evidence shows that such research support establishments generate high-quality scientific research, innovation and collaborations.^23^

We investigated three research publication metrics: (a) authorship positions (first and last authors) as an indicator of research effort and prestige,^24 25^ (b) research publication outputs as a measure of research productivity^26 27^ and (c) citation counts as an indicator of the quality and impact of research in different disciplines,^6 7^ especially in Science, Technology, Engineering and Mathematics (STEM) subjects including medicine.^8^ We analysed these publication metrics against three protected characteristics, that is, ethnicity, sex and age of authors affiliated with our BRC. Doing this study was imperative as there is limited understanding of and evidence on the diversity of authors of scientific research,^28^ especially in biomedical and translational health research.^29^ In addition, this study is important as it provides benchmark evidence and highlights methodological challenges in research on the diversity of scientific authorship.^30^

### Author ethnicity

We found a majority of white authors in both the first and last authorship categories in BRC2, BRC3 and the total study period (table 1), which is common in high-income and developed countries like the US, the UK and Canada.^31^ However, we found positive trends in the absolute number of BAME authors in both first and last authorship categories in the BRC3 period compared with the BRC2 period, but the proportions were not statistically significantly different between the two BRC periods. The proportions of BAME first and last authors in the total study period (2012–2022) were slightly lower than the proportions of non-white authors reported by others.^31^ However, these differences could be due to the author’s institution and country, as our findings are related to the NIHR Oxford BRC in England, while findings reported by Chander *et al*^31^ relate to different institutions in different countries, including the UK.

Our analysis of publication outputs by author ethnicity showed that nearly nine out of 10 publications were published by white authors, which was due to the greater number of white researchers in both BRC funding periods. Interestingly, our findings revealed a positive trend showing a slightly higher but statistically not significant increase in publications by BAME authors compared with the increase in publications by white authors (table 2, figure 4).

Our analysis of citations per publication by author ethnicity (figure 6) showed differences in citations of publications by white and BAME authors, which could be due to biases in citations that occur because of several factors such as the author’s sex/gender, ethnicity/race and country/nationality.^32–34^

### Author sex

Our analysis showed statistically significant differences between male and female authors in first and last authorship positions, as reported by earlier studies.^9 35 36^ We found greater proportions of male authors in the most prestigious authorship position i.e., last author in both BRC2 and BRC3 periods (table 1). These findings are not surprising as similar disparities have been reported by other researchers.^9 29^ These findings suggest that the gender gap in authorships remains to be narrowed down.^37–39^ We also found some positive trends showing an increase in the absolute number of female authors as first and last authors in the BRC3 period compared with the BRC2 period, but it was lower than the increase in male first and last authors in the BRC3 period (table 1, figure 3). Positive trends in authorship equity by author’s sex are also reported by others.^36^

Our findings of publication outputs by author sex also revealed a positive trend showing an increase in publications by female authors in the BRC3 period compared with the BRC2 period (table 2, figure 4), but the increase was lower than the proportional increase in publications by male authors. The under-representation of female authors in biomedical research publications in the UK has also been reported by earlier studies.^9 13^ Our earlier research on gender equity in the authorship of scientific publications had shown the overall proportion of female authors lower than male authors and significant increasing trends of female first, last and corresponding authors, while the proportions of male and female last authors were similar to their respective proportions as principal investigators in our BRC.^9^ A recent report on gender equality in research and innovation by Elsevier^12^ showed that on average, women researchers publish fewer publications than men in every country participating in the study, regardless of authorship position. The Elsevier report showed the least difference in the number of publications by women compared with men among first authors and the biggest difference among all other authorship categories.^12^

Our findings illustrate and confirm profound author diversity biases in both publication outputs and citation counts, as reported by others.^40 41^ These biases suggest a lack of EDI at the author level.^42^ There are several factors that could contribute to publication bias, such as the demographic homophily and relations between the editors, reviewers and authors,^43 44^ especially in the biosciences.^45^ This evidence implies that editors and reviewers prefer authors of the same sex/gender, ethnicity and nationality.^44 45^ Some earlier research concludes that overall male researchers are more productive than female researchers, but the differences in publications are affected by a combination of factors such as the age and position of the researcher, access to research funding and obtaining prestigious research grants, opportunities to become an independent researcher, and the research environment.^46^ Earlier research also shows that male researchers, especially last (senior) authors, are older in chronological age, have longer academic age and occupy higher academic positions.^47 48^ These factors are associated with many advantages such as access to research funding, research networks and collaborations and a high-impact research profile and publication portfolio.^49^ Conversely, there are few female scientists at higher positions and they are often less in prestigious authorship positions compared with male researchers.^1 9^ In addition, there are widening disparities in research projects,^50 51^ clinical-trial leadership^52^ and innovation and creativity due to researchers’ sex, as evident by global patenting trends showing women comprising only 23% of all applicants.^38^ These factors have a negative impact on women scientists’ research performance,^53^ scientific publication productivity^46^ and research impact.^54 55^ Women scientists therefore need guidance, support and mentoring and better research funding opportunities to excel in scientific research and innovation.^56^ However, we do not know the actual determinants of differences in publication outputs by researchers included in our sample. This aspect needs further exploration.

Our findings also show statistically significant differences in citation counts by the author’s sex, showing overall higher proportions of citations of publications by male authors compared with citations of female authors’ publications in both BRC periods (table 2). This is not surprising as similar trends in citations by author’s sex are reported by others.^25 57^ However, our analysis of the cumulative citations per publication over the total period of our study (2012–2022) and in the BRC3 period revealed that female authors’ publications were cited on average more than male authors’ publications (figure 6). These findings indicate a positive trend and support earlier findings that female scientists publish less but could get higher citations,^58^ indicating women scientists’ excellence in research and innovation.^59^

Our findings corroborate the body of research that shows citation inequality in medical sciences^60 61^ and a greater percentage of citations accumulated by a small group of elite scientists,^61^ which might be because of the Matthew effect on their research.^62 63^ However, the citation picture is more complex and the association between research productivity, quality and impact of research is not specific to the author’s sex only.^46^ There are several contributing factors such as unethical citation practices e.g., self-citation, journal prestige,^60 64^ research policies and cultural norms.^65^ These factors need to be explored and considered for a fairer assessment of EDI in research.

### Author age

Several bibliometric studies have focused on the association between the author’s age and publication metrics.^49 66^ For example, the author’s age and authorship positions,^67^ the author’s age and publication productivity,^48 68^ and the author’s age and citations.^48 66^ We studied the author’s age and trends in three publication metrics, that is, authorship positions, publication outputs and citation counts. We used the chronological age of authors, whereas most studies have used the author’s academic age as a proxy of the author’s actual age.^47 48^ We found statistically significant differences in first and last authorship positions by the author’s age in both the BRC2 and BRC3 periods as well as in the total study period (2012–2022) (table 1; Figure 2). Our findings showed that authorship positions are influenced by the author’s age and the relationship between the author’s age and first and last authorship positions is quadratic and non-linear.^67 69^ Our findings also showed that first authors are more likely to be young researchers, while the last authors are highly likely to be old researchers.^48 69^ However, the relationship between the author’s age and authorship positions is complex because the authorship position is determined and influenced by several factors such as the author’s academic age, experience, rank, role and other factors like the research team and network, academic discipline, institutional policies, and the authorship order culture in academic disciplines and research institutions.^47 70–72^

Our findings also show statistically significant differences in publication outputs by authors of different age groups in the BRC2 and BRC3 periods and the total study period. We found that authors aged 50–59 years and 40–49 years were the first and second most productive authors, who published the first and second highest proportions of publications, respectively, in the total study period (table 2). These findings are not surprising as earlier research shows that middle-aged authors are most likely to publish higher numbers of publications than authors of any other age.^48^ Because of the higher publication productivity, the author age of 40–59 years has been called the golden age of authors^73^ and we found that authors of this age group produced the highest proportion of publications in BRC2 (64.5%), BRC3 (69%) and the total period of our study (68%) (table 2). We also found that authors aged under 40 years produced the smallest proportion of publications, as reported by others.^48 68 69^ Young early-career researchers are more likely to produce a smaller number of publications because they face several challenges, such as short-term research contracts, lack of research training, mentorship and infrastructural support, often working alone on research projects with limited protected time, and encountering barriers in research publication and funding.^74^

Some earlier studies have suggested that publication productivity of authors does not decline in older age.^48 75^ However, we found declining trends in publications output by authors aged 60 and above, as reported by others.^69^ The declining trend in publication productivity of older authors could be due to various reasons, such as a higher pressure to acquire research funding and less pressure to write research articles^76^ and a change in the nature of research activity and focus to some other scholarly work.^75^ Overall, our findings show an inverted U-shaped concave relationship between the author’s age and research publication outputs,^77^ which suggests that publication productivity increases with author age, plateaus in the 50s and then declines in the 60s and onwards.^48 69 78^ However, this type of relationship between the author’s age and publication productivity has been contradicted by some earlier studies, which suggest that this pattern applies to some researchers but does not hold universally.^68 79^ These conflicting findings suggest that further research is needed.

The relationship between the author’s age and citations of scientific publications is also complex. Our findings show a curvilinear relationship between the author age and total citations. We found that authors aged 50–59 years had the highest proportion of total citations compared to authors of any other age group in BRC2, BRC3 and the total period of our study (table 2). The age of publication usually determines the number of citations; thus, older publications have higher citations than the newer publications.^66^ Very interestingly, citations of BRC3 publications of young authors (aged less than 50 years) increased manifold, while citations of publications by older authors (aged 50 years and above) decreased despite having the same publication age (BRC3, 2017–2022) (figure 5). Higher citations of recent publications could be due to various factors,^80^ which are related to the author, the journal and/or the publication.^81^ For example, the content of the publication; high importance, innovation and growth of the topic; the impact of research and accessibility (eg, open access publication); and the structural context of research, like publications from large prestigious research institutions.^82 83^ However, we could not be certain about the actual reasons for the differences in citations of publications of the same age in our study because of the limitations of our data. This issue needs to be explored in future research.

More interestingly, we found that authors aged 60–64 years had the highest average citations per publication in BRC2, BRC3 and the total study period (figure 6). Higher citations of publications by older authors could be due to several factors such as they are usually senior researchers and hold highly advantageous positions, for example, consultants and research group leaders leading several projects. They might be well-known authorities locally, nationally and perhaps even globally. Therefore, their publications from their local research group and/or from a national or an international collaboration are highly likely to get quick and higher visibility and accessibility soon after the publication; hence, they get quick attention and more citations.^84–86^ Our BRC is one of the world-famous centres for translational and biomedical research and our senior researchers work on cutting edge techniques and procedures in healthcare, which might have contributed to higher citations of publications of our senior researchers. However, this issue needs further investigation.

Our study provides evidence of widening author-level disparities in authorship of scientific publications in translational and medical sciences.^37 61^ Author level disparities in scientific publications are determined by a variety of complex factors such as the research environment, funding frameworks, research collaborations and networks, and characteristics of fields/disciplines.^87^ These disparities therefore need to be judged in a broader context at the national, sectoral, organisational, higher education, research funding and disciplinary levels. At the national level, the ethnic profile of the population in England shows that the white ethnic population comprises the highest proportion of the total population (85% and 81% in the 2011 and 2021 censuses, respectively) and the ethnic minorities comprise a smaller proportion of the population.^88^ Our findings about the ethnic background of the first and last authors (table 1) are reflective of these national-level ethnic proportions. In addition, the diversity in higher education staff data shows that a vast majority of professors are male (68% vs 32% female) and white (86%) (white male 53% and white female 26%) while only 14% of professors are of ethnic minority backgrounds.^89^ Moreover, employment in professional occupations in STEM disciplines is dominated by male professionals (75% men versus 25% women).^90^ These disparities in the higher education sector and professional occupations in STEM fields are reflected in our findings about the authors’ sex and ethnicity. Furthermore, in the healthcare sector, there are 69% white clinicians and 31% Asian clinicians (of these, 43.5% are associate specialists and 30% are consultants).^91^ However, these proportions of clinicians of ethnic minority backgrounds are not depicted in the authorship positions in our study. It is also imperative to look at the research employment diversity data.

Our BRC is a non-recruiting research centre. It is a partnership between the University of Oxford and Oxford University Hospitals NHS Foundation Trust. Hence researchers funded by our BRC are employees of either the university or the hospital trust. Staff diversity data of the University of Oxford shows more male academics (65%) and researchers (53%) than their female counterparts (35% academics and 47% researchers); and about 68% of academics and 54% of researchers are white compared with BAME academics (11%) and researchers (27%), whereas data about the ethnicity of 21% of academics and 19% of researchers is not available.^92^ A similar picture of staff diversity is present at the Oxford University Hospitals NHS Trust where clinical staff is 70% white and about 30% BAME.^93^ Our findings showing disparities in publication metrics by the author’s ethnicity, sex and age have roots in the broader contexts mentioned above. In addition, we must also consider the broader funding mechanisms. Our BRC is funded by the NIHR, and their award funding data shows that only 12% of applicants of ethnic minority backgrounds get research funding compared with 88% of white applicants, and 64% of funding is awarded to researchers aged 40 and 59 years, while the proportion of research awards by researcher’s sex is almost similar (61% women and 59% men).^94^ This data suggests that applicants who are younger in age and belong to ethnic minority backgrounds are less likely to secure research funding necessary to pursue their research pathways. All this evidence suggests that there is a need for proactive initiatives to facilitate, support, promote and provide fairer and equitable opportunities to disadvantaged researchers who are young, women and belong to ethnic minority backgrounds.^19 95^

Tackling these inequalities requires reinforcing institutional commitments to EDI in research at recruitment, training, career progression, authorship and funding levels through policies, programmes, training, retention and promotion of researchers of different ethnic backgrounds, age groups and sexes/genders.^96–98^ Such actions would require appropriate and proactive efforts at different levels including employers, research organisations and research funders.^99^ This would need strategic initiatives and policies such as the EDI strategies in research institutions and funding organisations. Some positive initiatives have already been taken such as the formulation of EDI strategies by our BRC^100^ and Oxford University Hospitals NHS Foundation Trust^101^ and the EDI strategic plan by the University of Oxford.^102^ The NIHR, our BRC’s funder, also has an EDI strategy.^3^ However, these strategies would take some time to narrow down the disparities in research. In order to achieve EDI in scientific authorship and research workforce at the NIHR BRC level, it is imperative to address structural inequalities^3 103^ through systematic investigation of factors such as recruitment, retention, training, career progression and promotion, and research funding barriers.^5 104^ Because these factors have a great potential to contribute to inequalities and exclusions of researchers at different levels in the NIHR BRCs.^105^ Moreover, for tracking progress on EDI, it would be imperative to collect researcher-level diversity data, including data on protected characteristics at the time of recruitment as well as data on publication metrics for reporting to the research funders such as the NIHR. It is also imperative that the NIHR BRCs work with under-represented groups of researchers, like women, ethnic minority and young researchers, and set support systems for them to address gaps in EDI in research and authorship.

### Study limitations

This study is not without limitations and challenges. The main limitation is the use of self-reported data on protected characteristics, which was not specifically collected for tracking diversity of authors (researchers) funded by our BRC, but it was provided voluntarily by researchers themselves during the recruitment process. The HR Department of the University of Oxford collated the data and provided it to us in an aggregated form. There is therefore a possibility that there was no data on the analysed protected characteristics of some researchers; thus resulting in their exclusion from the analysis. Nonetheless, we analysed a large data set that included three protected characteristics of BRC funded authors (476 unique researchers) and three important publication metrics covering about a decade (2012 to 2022).

It is also pointed out that we did not include the first funding period of our BRC (BRC1, 2007–2012) because the relevant data were not collected at this point. This is because reporting data on protected characteristics is not mandatory but voluntary^1^ and acquiring such data is difficult and complicated.^5^

For the NIHR BRCs, assessing and reporting diversity in biomedical scientific authorship is challenging because they do not directly employ researchers, and therefore they have to rely on partner organisations for researchers related HR data. These arrangements create limitations such as the provision of aggregated data on the ethnicity, sex/gender and age of researchers, which create limitations to assessing the actual representation of some groups. For example, we had aggregated data on ethnicity as white and BAME authors; therefore, there were no data on the exact proportions of authors who were of Black, Asian, or other ethnic minority backgrounds included in the BAME ethnic group. This issue could be addressed by collecting the author-level demographic data, including protected characteristics data, at the BRC level.

Another important limitation is that this baseline feasibility study is based on a bibliometric analysis linked with self-reported data on protected characteristics of authors. This approach does not fully help in understanding and unearthing the actual reasons for the under-representation of authors of certain protected characteristics. These data also do not help to identify the determinants of disparities in publication outputs and citation counts due to the author characteristics. Therefore, further research is required to explore these issues in detail.

## Conclusion

Our study provides important baseline evidence on the diversity of authorship, using three protected characteristics, that is, ethnicity, sex and age of authors, in biomedical and translational health research funded by the NIHR Oxford BRC. Our findings reveal significant inequalities in authorship positions, publication outputs and citation counts at the author level.

In this study, we were reliant on researchers’ self-reported protected characteristics, that is, ethnicity, sex and age data provided as part of the recruitment process and collated by the HR Department of the University of Oxford. Improved collection of such data would provide important evidence to determine the denominator of researchers with such protected characteristics being recruited and facilitate clearer actions to address potential recruitment bias of researchers. In addition, collecting diversity data of researchers and authors of publications funded by NIHR BRCs would be helpful in tracking progress on EDI in biomedical and translational health research. Further research is required to improve data quality and ways of capturing diversity of scientific research authorship.

## Data Availability

Data are available upon reasonable request.
