# Supplementary material for: Trends in diversity of scientific authorship by ethnicity, sex and age of authors in a National Institute for Health and Care Research Biomedical Research Centre in England: a retrospective longitudinal bibliometric study
Source: BMJ Open. 2026 Oct 15;16(10):e115677. doi: 10.1136/bmjopen-2025-115677 (PMC13691827; doi:10.1136/bmjopen-2025-115677)
Supplement: Supplementary file 1 [file bmjopen-2025-115677.reviewer_comments.pdf]

PEER REVIEW HISTORY

BMJ Open publishes all reviews undertaken for accepted manuscripts. Reviewers are asked to complete a checklist review form and are provided with free text boxes to elaborate on their assessment. These free text comments are reproduced below.

ARTICLE DETAILS

Title (Provisional)

Trends in diversity of scientific authorship by ethnicity, sex and age of authors in a National Institute for Health and Care Research Biomedical Research Centre in England: A retrospective longitudinal bibliometric study

Authors

Henderson, Lorna R; Barrio, F. Eugenio; Saunders, David; Coxall, Owen; Kiparoglou, Vasiliki; Kalpazidou Schmidt, Evanthia; Shah, Syed Ghulam Sarwar

---

VERSION 1 - REVIEW

**Reviewer**	**1**
**Name**	**Balas, Andrew**
**Affiliation**	**Augusta University**
**Date**	**11-Jan-2026**
**COI**	**None**

Obviously, there are major problems in the way the research enterprise involves various gender, age, and minority groups. Better understanding of the situation could lead to some targeted actions.

The study presented in this article has a meritorious starting question, but what follows is a diffuse and somewhat unfocused analysis without clear actionable recommendations emerging.

Apparently, the authors of this study have access to a very important and informative database relevant to the objectives of this study. However, the database collected by the authors could be used much more effectively. A few specific shortcomings:

The conclusions as presented are largely generalities already known from other studies and common experience.

The analyzed NIHR Oxford BRC is a very specific and in many ways privilege population of researchers. The authors should provide explanation for the generalizability of their results.

It would be important to know how these results are comparing the other countries and other areas.

It is unclear whether the shortcomings in participation are the results of hiring practices or come from fewer opportunities provided to certain people.

The study focuses on biomedical and translational health research funded by the NIHR Oxford BRC. This evaluated organization is also the financial sponsor of this study, a conflicting relationship that needs to be explained.

In summary, the authors should turn their study into problem-solving research i.e., effectively helping other people elsewhere by providing novel insight and actionable recommendations.

Reviewer **2**

Name **Bradley, Elizabeth**

Affiliation **Yale University, Vassar College**

Date **06-Feb-2026**

COI **None**

The findings are interesting and useful, and I appreciate the care in presenting them all. I would find the publication would have great impact with the following revisions:

1. Try to be more succinct; I could see this as a brief report in fact with tables to help give the data and remove data from the text. It is highly descriptive and gets overwhelming to read.

2. Do not repeat the findings in the discussion. Just give them once--in results. The discussion should be used more to interpret the results.

3. Main concern for me remained unanswered: is this because journals discriminate or does it merely reflect the differences in larger social forces on men and women so fewer women to begin with in these fields, etc. Hard to know what to make of the data without more understanding of this.

Reviewer **3**

Name **Morgan, Ben**

Affiliation **National Institute for Health Research Central Commissioning Facility**

Date **17-Feb-2026**

COI **I work for the National Institute for Health and Care Research (NIHR).**

This is an interesting study which builds on wider literature of trends in scientific authorship. I did not have any major concerns but thought some of the following items could have been addressed:

• Is there clarity on the characteristics of the 476 researchers to provide context to the results. There is content in the discussion on UK wide census results, but it is not clear what the characteristics of the authors were to begin with.

• In terms of ethnicity, it is uncommon to see simply white and BAME reported. NIHR reporting would typically break down BAME into Black, Asian, Mixed (as per the Diversity Data Report 21-22) – is there opportunity to examine if there are any differences within the currently reported BAME group?

• Is there a reason why BRC 1 data were not included?

There were also a number of inconsistencies and typos to address:

• Table 1 missing 'last author' under BRC3 heading

• Table 1 – BRC age range percentages come to 99% for first and last authors

• Table 1- total study period combined percentages come to 102%

• As above for table 2 (BRC 2 age)

• While the authors state they are examining sex there is interchangeable use of sex and gender later in the paper.

• Page 21 row 32 – 'demographic haemophilia' is presumably supposed to be 'demographic homophily'

• Page 23 row 53 'out findings' should be 'our findings'

• Page 27 row 34 'cutting age' techniques presumably should be 'cutting edge'

• Page 28 – the discussion on percentage of white vs BAME academics and researchers do not total 100%

VERSION 1 - AUTHOR RESPONSE

Reviewer: 1 Dr. Andrew Balas, Augusta University
Comments to the Author:

Obviously, there are major problems in the way the research enterprise involves various gender, age, and minority groups. Better understanding of the situation could lead to some targeted actions.

Authors response: This study sets out to determine a baseline using novel techniques. It builds on a number of studies which have focussed primarily only on gender equity in NIHR BRC Oxford and Medical Sciences Division of the University of Oxford (please see references # 1, 5, 9).

The study presented in this article has a meritorious starting question, but what follows is a diffuse and somewhat unfocused analysis without clear actionable recommendations emerging.

Authors response: Thanks for your constructive feedback. The aim of the study is to set a baseline understanding. This is the first cohort, and so actionable recommendations could be to work with BAME researchers and set support for those underrepresented groups. Please see our recommendations on pages 28-29.

Apparently, the authors of this study have access to a very important and informative database relevant to the objectives of this study. However, the database collected by the authors could be used much more effectively.
Authors response: Thanks for your suggestions. That is why we have analysed this publication database.

<u>A few specific shortcomings:</u>
The conclusions as presented are largely generalities already known from other studies and common experience.

Authors response: Thanks for your comments. We have revised the manuscript. This study is different as it is written from the perspective of a BRC funded by NIHR who have set key targets linked to future funding. To our knowledge no other data has been published in a BRC setting. The methods could therefore be applied to other BRCS. BRC is focusing now on underserved groups such as female, ethnic minority and young researchers.

The analyzed NIHR Oxford BRC is a very specific and in many ways privilege population of researchers. The authors should provide explanation for the generalizability of their results.

Authors response: Implications of the generalisability of our findings are reported in the discussion on page #. This study builds on previous research on gender equity and other research at the University of Oxford (please see references # 1, 5, 9, 19). NIHR BRCs are commissioned across England, and the methods could be generlisable to other NIHR BRC settings. The discussion flags this. BRCs are not employers and so this study has sought to use the database to draw out key findings.

It would be important to know how these results are comparing the other countries and other areas.

Authors response: We have compared our findings with some other studies conducted in other countries such as the UK, the US, Canada and France (please see references 13, 14, 31, 35, 37, 39, 43, 47, 59 and 60) and see the relevant discussion on page numbers #.

It is unclear whether the shortcomings in participation are the results of hiring practices or come from fewer opportunities provided to certain people.

Authors response: Thanks for highlighting this critical issue. However, this is a baseline study, and a bibliographic analysis. So further qualitative research would be required to explore this in detail.

The study focuses on biomedical and translational health research funded by the NIHR Oxford BRC. This evaluated organization is also the financial sponsor of this study, a conflicting relationship that needs to be explained.

Authors response: The study was driven by our funders requirement to demonstrate EDI at all levels in BRCs. Therefore, we were best placed to complete the study as we have access to the data and understanding of the context. The results can now be actioned at BRC level.

In summary, the authors should turn their study into problem-solving research i.e., effectively helping other people elsewhere by providing novel insight and actionable recommendations.
Authors response: the novel insights the findings from this study are highlighted and the highlights of this study what is new is also reported. The discussion and conclusion provide some suggestions for further research (see page 28 and 29).

Reviewer: 2: Dr. Elizabeth Bradley, Yale University
Comments to the Author:

The findings are interesting and useful, and I appreciate the care in presenting them all. I would find the publication would have great impact with the following revisions:

Authors response: Thanks for your feedback and comments which are highly appreciated

1. Try to be more succinct; I could see this as a brief report in fact with tables to help give the data and remove data from the text. It is highly descriptive and gets overwhelming to read.

Authors Response: Thanks for the suggestions we have revised the text and avoided repetition of data in tables and text.

2. Do not repeat the findings in the discussion. Just give them once--in results. The discussion should be used more to interpret the results.

Authors response: We have revised the text.

3. Main concern for me remained unanswered: is this because journals discriminate or does it merely reflect the differences in larger social forces on men

and women so fewer women to begin with in these fields, etc. Hard to know what to make of the data without more understanding of this.

Authors response: The aim of the study is set out a baseline understanding. This is the first study to our knowledge which has done this. Research demonstrates clear inequities for women in science however this is the first study to our knowledge to extend this to other protected characteristics e.g. age/ethnicity. Further research on systemic issues would be required to understand the drivers.

Reviewer: 3. Dr. Ben Morgan, National Institute for Health Research
Comments to the Author:

This is an interesting study which builds on wider literature of trends in scientific authorship. I did not have any major concerns but thought some of the following items could have been addressed:

Authors response: Thanks for your constructive comments and suggestions.

• Is there clarity on the characteristics of the 476 researchers to provide context to the results.

Authors response: NIHR BRCs are required to demonstrate diversity however as BRCs do not directly employ researchers this is challenging. In this scenario the majority of researchers are employed by the University of Oxford. As part of our annual reporting which is a contractual requirement to NIHR we do not collect demographic information and there are two different employers e.g. NHS Trusts and University of Oxford. The study therefore sets out a novel way of collecting this information indirectly and anonymously via the HR data and publications dataset. Only names no other characteristics. Reported in our annual reports submitted to the NIHR.

• There is content in the discussion on UK wide census results, but it is not clear what the characteristics of the authors were to begin with.

Authors response: We have talked about the census data etc to show the wider picture because the ethnicity or gender related issues are entrenched in the national fabric and must be considered in the broader context rather than at the BRC level. Our findings are reflective of these situations.

• In terms of ethnicity, it is uncommon to see simply white and BAME reported. NIHR reporting would typically break down BAME into Black, Asian, Mixed (as per the Diversity Data Report 21-22) – is there opportunity to examine if there are any differences within the currently reported BAME group?

Authors response: This is because our HR department provided data on ethnicity comprising two ethnic groups i.e. White and BAME. We do not have breakdown and there were perhaps low numbers in different ethnicities included in BAME group, which

could have raised the issue of identification of these researchers. We have therefore recommended that the author level diversity data should be collected at the BRC level.

• Is there a reason why BRC 1 data were not included?

Authors response: These data were not collected at this point (BRC1) and so not available. Hence, this period was excluded. We have highlighted this fact (see 'Data' on page 7).
There were also a number of inconsistencies and typos to address:

• Table 1 missing 'last author' under BRC3 heading

Authors response: Added 'last author'.

• Table 1 – BRC age range percentages come to 99% for first and last authors

Authors response: Corrected. It was due to rounding of decimals.

• Table 1- total study period combined percentages come to 102%.

Authors response: Corrected. It was due to rounding of decimals.

• As above for table 2 (BRC 2 age)

Authors response: Corrected. It was due to rounding of decimals.

• While the authors state they are examining sex there is interchangeable use of sex and gender later in the paper.

Authors response: Gender changed to sex.
• Page 21 row 32 – 'demographic haemophilia' is presumably supposed to be 'demographic homophily'.

Authors response: Corrected as 'demographic homophily'.

• Page 23 row 53 'out findings' should be 'our findings'

Authors response: Corrected 'Out' to 'Our'.

• Page 27 row 34 'cutting age' techniques presumably should be 'cutting edge'

Authors response: Corrected as 'cutting edge'

• Page 28 – the discussion on percentage of white vs BAME academics and researchers do not total 100%

Authors response: Checked and updated. It was due to non-declaration of the ethnicity by 21% of academics and 19% of researchers. This information has been added now.

VERSION 2 - REVIEW

**Reviewer**	**2**
**Name**	**Bradley, Elizabeth**
**Affiliation**	**Yale University, Vassar College**
**Date**	**11-Jul-2026**
**COI**	

I found the discussion too long--a repeat of the results. Also, two paras (beginning with discussion of bias) seem to lead the reader to make conclusions beyond the data in this manuscript and makes declarative statements about other literature we aer not reviewing, and I found that very distracting and made me question to bias of the authors. I would have preferred they stuck to discussing their findings and their implications, plausible explanations, but not para after para on other literature that supposes what these results may mean in a speculative way.

VERSION 2 - AUTHOR RESPONSE

Reviewer: 2 Dr. Elizabeth Bradley, Yale University Comments to the Author: I found the discussion too long--a repeat of the results. Also, two paras (beginning with discussion of bias) seem to lead the reader to make conclusions beyond the data in this manuscript and makes declarative statements about other literature we are not reviewing, and I found that very distracting and made me question to bias of the authors. I would have preferred.

Authors response: We have substantially edited the discussion section and edited our statements. However, it is imperative to mention that EDI needs to be assessed in the wider contexts. We have retained the literature on the factors that could affect the EDI but we have clearly stated that due to the limitations of our data we could not be certain about the reasons of disparities; therefore, further research is needed. Wider literature like the HESA data is used by the NIHR in their diversity data reports for the comparison purposes.
For example, see para 1 and 3 on page 21, para 2 on page 23, para 1 on page 24, para 1 on page 25 [Main document -clean copy

Reviewer: 2 If you have selected 'Yes' above, please provide details of any competing interests.

Authors response: Please see the competing interests on page 30 of the main document (clean copy)
